# Malaria in school-age children in Africa: an increasingly important challenge

**DOI:** 10.1111/tmi.12374

**Published:** 2014-08-22

**Authors:** Joaniter Nankabirwa, Simon J Brooker, Sian E Clarke, Deepika Fernando, Caroline W Gitonga, David Schellenberg, Brian Greenwood

**Affiliations:** 1 Makerere University College of Health SciencesKampala, Uganda; 2 Faculty of Infectious and Tropical Diseases, London School of Hygiene & Tropical MedicineLondon, UK; 3 Department of Parasitology, Faculty of Medicine, University of ColomboColombo, Sri Lanka; 4 MEASURE Evaluation, ICF InternationalNairobi, Kenya

**Keywords:** malaria, school-age children, Africa

## Abstract

School-age children have attracted relatively little attention as a group in need of special measures to protect them against malaria. However, increasing success in lowering the level of malaria transmission in many previously highly endemic areas will result in children acquiring immunity to malaria later in life than has been the case in the past. Thus, it can be anticipated that in the coming years there will be an increase in the incidence of both uncomplicated and severe malaria in school-age children in many previously highly endemic areas. In this review, which focuses primarily on Africa, recent data on the prevalence of malaria parasitaemia and on the incidence of clinical malaria in African school-age children are presented and evidence that malaria adversely effects school performance is reviewed. Long-lasting insecticide treated bednets (LLIN) are an effective method of malaria control but several studies have shown that school-age children use LLINs less frequently than other population groups. Antimalarial drugs are being used in different ways to control malaria in school-age children including screening and treatment and intermittent preventive treatment. Some studies of chemoprevention in school-age children have shown reductions in anaemia and improved school performance but this has not been the case in all trials and more research is needed to identify the situations in which chemoprevention is likely to be most effective and, in these situations, which type of intervention should be used. In the longer term, malaria vaccines may have an important role in protecting this important section of the community from malaria. Regardless of the control approach selected, it is important this is incorporated into the overall programme of measures being undertaken to enhance the health of African school-age children.

## Introduction

The age distribution of cases of malaria is influenced strongly by the intensity of malaria transmission. In areas where the population is exposed only occasionally to an infectious bite, malaria occurs in subjects of all ages, often most frequently in adults who have an occupational risk. In contrast, in areas of high transmission, the main burden of malaria, including nearly all malaria deaths, is in young children (Snow & Marsh 2002; Carneiro *et al.* 2010). Until recently, malaria transmission in most malaria endemic areas of sub-Saharan Africa was moderate or high and control measures consequently focussed on the protection of young children and pregnant women. However, enhanced control efforts have recently reduced the level of malaria transmission in many parts of sub-Saharan Africa (O'Meara *et al.* 2010; Noor *et al.* 2014) and in many areas where transmission was previously hyper or holo-endemic (malaria parasite prevalence in children aged 2–10 years)(PfPR_2–10_ > 50%) it has become mesoendemic. As a consequence children are acquiring immunity to malaria more gradually than in the past and clinical attacks, sometimes severe, are occurring in school-age children more frequently. However, the epidemiology and management of malaria in school-age children has, until recently, received little attention (Brooker *et al.* 2008; Brooker 2009). In this review, information on the current burden of malaria in African school-age children is presented and novel approaches that are being explored to control malaria in this increasingly important group are reviewed.

## Methods

A structured review was undertaken of published literature on malaria in school-age children cited in PubMed using combinations of the following key terms: malaria, *Plasmodium*, anaemia, cognition, education, school children, schools, children, control, bed nets, treatment. This review was supplemented by consideration of additional papers generated by these searches or otherwise known to the authors. The review has focused primarily on studies from Africa published within the past 20 years, although other references are included when these are relevant to the current situation. We define school-age children as those aged between 5 and 14 years, although some studies have included children covering a wider age range.

### The prevalence of malaria in African school-age children

**Fig 1:**
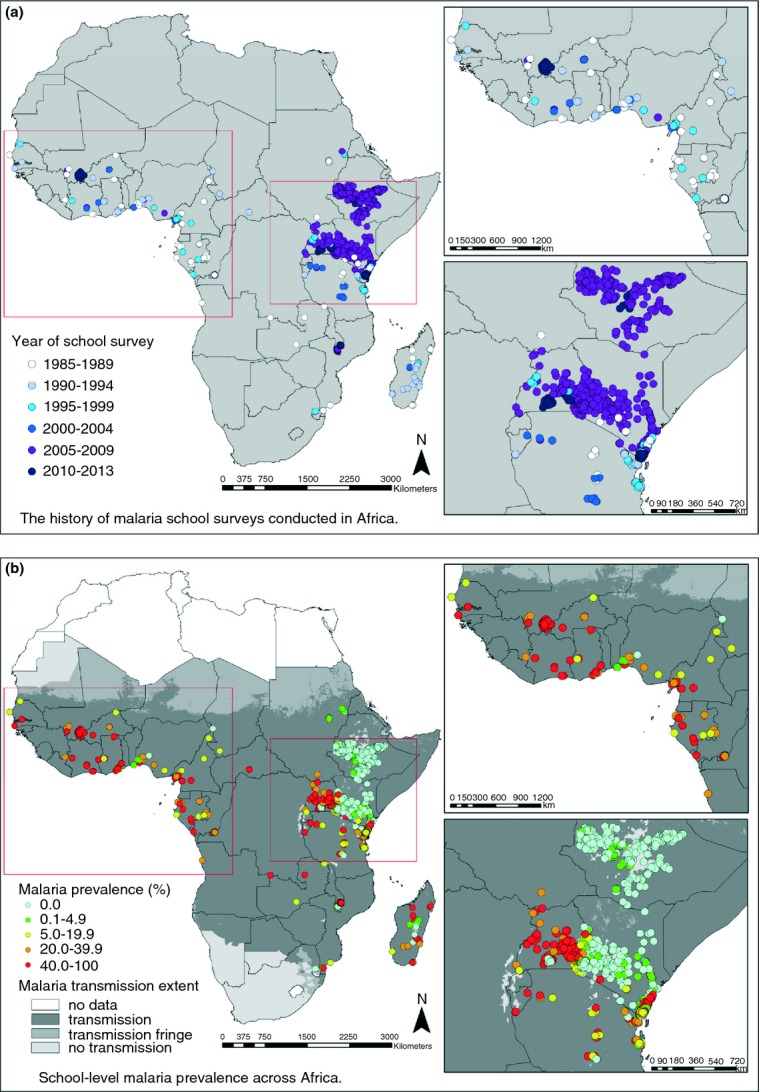
Figure1a shows the frequency with which malaria surveys have been undertaken in school-age children over time and Figure1b the prevalence rate observed in school-age children by geographical area based on data gathered and provided by the MAP project (www.map.ox.ac.uk).

In 2010, it was estimated that over 500 million school-age children were at risk of malaria infection, 200 million in sub-Saharan Africa (Table1) (Gething *et al.* 2011). Only a small number of surveys of the prevalence of *Plasmodium falciparum* in school-age African children have been undertaken, although there has been an increase in the number and geographical extent of schools surveyed in East Africa in recent years (Figure1a). Figure1b shows the prevalence rate observed in school-age children by geographical area based on data gathered and provided by the Malaria Atlas Project (www.map.ox.ac.uk).

**Table 1:** Estimated school-age (5–14 years) population at risk of *Plasmodium falciparum* malaria in 2010 (figures in millions). Adapted from Gething *et al*. (2011)

Region	Unstable risk	Stable risk	Total
America	11.80	6.41	18.21
Africa plus Yemen and Saudi Arabia	11.19	200.88	212.06
Central, South and East Asia	205.43	132.28	337.71
World	228.41	339.57	567.99

As expected, the prevalence of *P. falciparum* in African school-age children varies widely from area to area, even within the same country, depending on the level of transmission (Table2). For example, in Uganda 14–64% of school-age children were parasitaemic at any one time, with the parasite rate depending upon transmission setting and season (Nankabirwa *et al.* 2010; Pullan *et al.* 2010; Nankabirwa *et al.* 2013; Kabatereine *et al.* 2011). In neighbouring Kenya, the prevalence of infection in school-age children also varied widely from place to place; a country wide survey conducted in 480 Kenyan schools between September 2008 and March 2010 found an overall prevalence of malaria parasitaemia of 4%, but this ranged from 0 to 71% between schools (Gitonga *et al.* 2010, 2012). In Senegal, The Gambia, and Mauritania, the prevalence of infection in school-age children ranged from 5 to 50% (Dia *et al.* 2008; Ouldabdallahi *et al.* 2011; Clarke *et al.* 2012; Oduro *et al.* 2013) with prevalence rates showing marked seasonal variation. Surveys conducted in school-age children in the South West province of Cameroon (Nkuo Akenji *et al.* 2002; Kimbi *et al.* 2005, 2013; Achidi *et al.* 2008) found parasite rates in school age children of about 50%, with a lower rate among those living higher up Mount Cameroon.

**Table 2:** Recent studies on the prevalence of malaria parasitaemia among school-age children

Country	Transmission setting	Age range (years)	Year of survey	Estimated prevalence (%)	Source
East Africa					
Uganda	High	8–14	2008	51	Nankabirwa *et al*. (2010)
	High	5–9	2008	64	Pullan *et al*. (2010)
	Moderate	10–12	2009–2010	46	Kabatereine *et al*. (2011)
	High	6–14	2011	30	Nankabirwa *et al*. (2013)
	High	6–15	2012	56.5	Uganda Malaria Surveillance Project (un-published)
	Moderate	6–15	2012	16	Uganda Malaria Surveillance Project (un-published)
	Low	6–15	2012	14	Uganda Malaria Surveillance Project (un-published)
Kenya	High	8–14	2002	23	Clarke *et al*. (2004)
	Epidemic prone	8–14	2002	47*	Clarke *et al*. (2004)
	High	5–18	2005–2006	41	Clarke *et al*. (2008)
	High	5–18	2008–2010	18	Gitonga *et al*. (2012)
	Seasonal	5–18	2008–2010	2	Gitonga *et al*. (2012)
	Moderate	5–18	2008–2010	3	Gitonga *et al*. (2012)
	Low	5–18	2008–2010	<1	Gitonga *et al*. (2012)
Tanzania	High	Mean 7.96	2005	35	Mboera *et al*. (2011)
	High	0.5–14	2011	9–23	West *et al*. (2013)
West Africa					
Senegal	Seasonal	≤9	2004–2005	9	Dia *et al*. (2008)
	Seasonal	6–14	2004–2006	0.9	Ouldabdallahi *et al*. (2011)
	Moderate-high seasonal	7–14	2011	54	Clarke *et al*. (2012)
The Gambia	Seasonal	6–12	2008–2009	17	Oduro *et al*. (2013)
	Seasonal	4–21	2011	14	Takem *et al*. (2013)
Cote d'Ivoire	High	5–9	1998–1999	66	Assi *et al*. (2013)
	High	6–10	2001–2002	67	Raso *et al*. (2005)
	High	6–14	2006–2007	58	Rohner *et al*. (2010)
Mali	High, seasonal	6–14	2007–2008	42	Thuilliez *et al*. (2010)
	High, seasonal	7–14	2011	83	Clarke *et al*. (2012)
Nigeria	High	8–16	2007–2008	26	Ojurongbe *et al*. (2011)
Central Africa					
Cameroon	High	2–11	2002	30	Nkuo Akenji *et al*. (2002)
	High	4–16	2006	40	Wanji *et al*. (2008)
	High	4–12	2007	59	Achidi *et al*. (2008)
	High	4–15	2009	34	Kimbi *et al*. (2013)
Congo Brazzaville	High	1–9	2010	16	Ibara-Okabande *et al*. (2012)
Equatorial Guinea	High	5–9	2009–2010	40.0	Rehman *et al*. (2011)
	High	10–14	2009–2010	42.0	Rehman *et al*. (2011)
Other parts of Africa					
Ethiopia	Low	5–16	2009	0–15	Ashton *et al*. (2011)
Yemen	Low	6–11	2001	13	Bin Mohanna *et al*. (2007)
Somalia	Low	5–14	2007	20.5	Noor *et al*. (2008)
Mozambique	High	5–7	2002–2003	48.1	Mabunda *et al*. (2008)
Malawi	High	5–9	2009–2010	53.0	Rehman *et al*. (2011)
	High	10–14	2009–2010	52.0	Rehman *et al*. (2011)

* Recorded during an outbreak.

### The clinical consequences of malaria in school-age African children

#### Mortality

The number of school-age children who die from malaria each year is not known. A study by Snow *et al*. (2003) estimated that, at that time, malaria was responsible for 214 000 deaths per year among Africa school-age children, representing up to 50% of all deaths among this age group. More recently, Murray *et al*. (2012), using a combination of vital registration data and verbal autopsy data, estimated that in 2010 6–9% of all malaria deaths occur in the 5–14 year age group, giving a figure in the range of 70–110 000 deaths per year.

#### Clinical attacks of malaria

The incidence of clinical attacks of malaria in school-age African children is poorly defined because this age group is not included routinely in household-based cluster surveys such as malaria indicator surveys, demographic health surveys or multiple indicator cluster surveys. Information on the current incidence of malaria in school-age children is derived mainly from World Health Organisation (WHO) estimates and from occasional school-based, active case detection studies. The former usually depend upon country-based surveillance systems which currently detect only about 10% of clinical cases, with the detection rate being lowest in countries with the highest numbers of malaria cases (WHO 2012). The most frequent sources of information on the burden of disease in school-age children are research studies but these vary largely in their methodology (Table3). In 2002, Clarke *et al*. (2004) found an incidence of clinical attacks of malaria in children aged 8–14 years of 0.47 attacks per year during an outbreak in Nandi, Kenya and an incidence of 0.23% in Bondo, a high transmission setting. In Tororo, Uganda, 7% of children aged 6–14 years experienced a clinical attack of malaria during 42 days of follow-up (Nankabirwa *et al.* 2010) and in 2011 an incidence of 0.34 clinical episodes of malaria per child per year was recorded in the same area (Nankabirwa *et al.* 2014). The incidence of clinical episodes of malaria in children enrolled in the control arm of a trial in Cote d'Ivoire was 39% during a 6-month period of follow-up (Rohner *et al.* 2010) and in a trial in Mali it was 56% during a 9-month period of follow-up (Barger *et al.* 2009). We are unaware of any systematic studies that have documented changes in the incidence of clinical malaria in school-age children over time.

**Table 3:** Recent, published reports of the incidence of malaria in school-age children

Location	Transmission setting	Year	Method	Follow-up period	Sample size	Age range years	Observed incidence	Calculated annual incidence*	Source
Year-round transmission									
Uganda	High perennial	2011	Active case detection through daily roll call†	12 months	740	6–14	83 episodes/242.7 child-years at risk	0.34 episodes/child/year	Nankabirwa *et al*. (2014)
Kenya	High perennial	2002	Active case detection by visiting children 2–3 times per week	11 weeks	276	8–14	0.005/child-weeks at risk	0.26/child/year	Clarke *et al*. (2004)
Kenya	Epidemic prone	2002	Active case detection by visiting children 2–3 times per week	11 weeks	330	8–14	0.029/child-weeks at risk	1.5/child/year during epidemic outbreak	Clarke *et al*. (2004)
Ghana	Moderate	2002	Active case detection through weekly visits	9 months	352	6–10	0.22–0.25/child/year	0.22–0.25/child/year	Dodoo *et al*. (2008)
Highly seasonal transmission									
Burkina Faso	High, seasonal	2003	Active case detection through daily visits	4 months	51	6–8	2.7/child-year at risk	2.7/child/year	Nebie *et al*. (2008)
					65	8–11	0.59/child-year at risk	0.59/child/year	
					65	11–15	0.37/child-year at risk	0.37/child/year	
Mali	High, Seasonal	2007–2008	Active case detection through monthly visits†	8 months	98	6–13	1.46/child-year at risk	1.46/child/year	Barger *et al*. (2009)
Gambia	Seasonal	2008–2009	Active case detection through weekly visits	22 weeks	439	6–15	0.004/child-week at risk	0.025/child/year	Ceesay *et al*. (2010)
Other									
Ethiopia	Low	2009–2011	Active case detection through weekly visits and passive detection of cases between the weekly visits	101 weeks	2075	5–14	110/2075 for 101 weeks	0.03/child/year	Loha and Lindtjørn (2012)

* Calculation of the annual incidence assumes uniform incidence throughout the year for areas of perennial transmission, In areas of highly seasonal transmission where transmission is limited to a few months each year, total annual incidence is assumed to equate to that measured during the period of observation.

† Data collected during an intervention trial; incidence data refer to observations in the control arm.

#### Anaemia

Anaemia is prevalent among school-age children in the tropics. Its aetiology is usually multi-factorial but it is likely that, in many communities, malaria plays an important role (Kassebaum *et al.* 2014). The strongest evidence that malaria is an important cause of anaemia in children comes from intervention studies, but these have focused largely on those under 5 years of age (Korenromp *et al.* 2004). An early study of the impact of indoor residual spraying (IRS) on individuals living in the Taveta-Pare area of Kenya and Tanzania found that the haemoglobin concentration increased by 13 g/l among children aged 5–9 years old as a consequence of an IRS programme (Draper 1960). More recently, Clarke *et al*. (2008) showed that intermittent preventive treatment (IPT) with sulfadoxine-pyrimethamine (SP) in combination with amodiaquine (AQ) significantly reduced the prevalence of anaemia during 12 months of follow-up in a large trial conducted in Kenyan schoolchildren. Similarly, Nankabirwa *et al*. (2010) demonstrated that IPT with SP + AQ or dihydroartemisinin-piperaquine (DHA-PQ) improved the haemoglobin concentration of Ugandan school-age children over a follow up period of 42 days and these investigators showed that monthly IPT with DHA-PQ given over 1 year significantly also reduced the risk of anaemia in children in the same age group (Nankabirwa *et al.* 2014).

### The educational consequences of malaria in school-age African children

An important reason underlying a recent interest in malaria in school-age children is the concern that malaria may interfere with a child's educational development.

#### Malaria and school-absenteeism

There is strong evidence that malaria is an important cause of school absenteeism. A study undertaken in Nigeria showed that malaria caused an average loss of three school days per episode (Erinoso & Bamgboye 1988). Evidence suggests that between 0.001 and 0.021 days are lost from school due to malaria per child per annum, accounting for between 2% and 8% of all episodes of absenteeism (Colbourne 1955; Trape *et al.* 1987, 1993). Malaria is thought to account for between 13% and 50% of the medical reasons for absenteeism from school. The educational impact of malaria is greater for primary school children than secondary school children: a study in Kenya found that malaria caused a loss of 11% and 4.3% of the school year for primary and secondary school students respectively (Leighton & Foster 1993). Another study, undertaken in the highlands of Kenya, estimated that during a malaria epidemic, malaria-related absenteeism in primary school pupils varied between 17% and 54% (Some 1994). The estimated annual loss of school days in Kenya due to malaria in 2000 was estimated to be 4–10 million days (Brooker *et al.* 2000).

#### Cognitive function

Several studies have shown that children who survive an episode of cerebral malaria may have residual impairment of cognition, speech, language and/or motor skills (Carter *et al.* 2005a,b, 2006; Boivin *et al.* 2007). In Côte d'Ivoire, cognitive defects persisted for at least 2 years after an episode of cerebral malaria (John *et al.* 2008) and similar findings were recorded in Ugandan children (Boivin *et al.* 2007). In Malawi, children with retinopathy-positive cerebral malaria had a persistent defect in language development (Boivin *et al.* 2011).

Cerebral malaria, a relatively uncommon outcome of malaria infection, is not a pre-requisite for cognitive impairment which may occur during the course of an uncomplicated clinical episode of malaria (Fernando *et al.* 2003a,b), and repeated episodes of uncomplicated malaria may have long-term effects (Fernando *et al.* 2003a,b). There is even some evidence that asymptomatic parasitaemia can impair cognitive function. In the Yemen, Al Serouri *et al*. (2000) showed that children with parasitaemia performed less effectively on formal cognitive testing than children without parasitaemia, even after adjusting for confounding factors. This was also the case in Uganda (Nankabirwa *et al.* 2013) and in Mali, although in Mali the effect was not as marked as in children with clinical malaria (Thuilliez *et al.* 2010). In Zambia, a strong association was found between exposure to malaria and cognitive skills and socio-emotional development in young children (mean age 74 months) (Fink *et al.* 2013).

The strongest evidence that malaria impairs cognitive function comes from intervention trials. In Sri Lanka, a randomized, placebo controlled, double-blind trial of chloroquine prophylaxis in children aged 6–12 years showed that educational attainment improved and that school absenteeism was reduced significantly (*P* < 0.0001) in children who took malaria prophylaxis (Fernando *et al.* 2006). In The Gambia, the educational achievement of children with an average of 17 years was better in those who had received malaria chemoprophylaxis during their first five years of life than in children who had received placebo (Jukes *et al.* 2006). In a more recent, larger, stratified, cluster-randomized, double-blind, placebo-controlled trial conducted in Kenya, IPT with SP + AQ significantly improved sustained attention of 10–12 year old schoolchildren (Clarke *et al.* 2008) and similar findings were obtained in a recent trial in school children in Mali (Clarke *et al.* 2013a,b).

### Treatment of malaria in school-age African children

In many parts of Africa, there are still geographical and financial barriers that prevent school-age children obtaining rapid access to diagnosis and treatment of malaria, and several approaches have been made to try to overcome these barriers.

#### Education

Schools can play a vital role in ensuring that their pupils understand the importance of obtaining rapid access to diagnosis and treatment of malaria by providing appropriate health education in school but, unfortunately, this is rarely part of the school curriculum. A content analysis of school text books in nine countries in Africa and Asia found that most included information on the mode of transmission of malaria and on the signs and symptoms of malaria but little about insecticide-treated nets (ITNs) or about the need for prompt and appropriate treatment of a clinical attack (Nonaka *et al.* 2012).

#### Diagnosis and treatment at school

Prompt and effective treatment of malaria can be enhanced by provision of treatment at school. In the past, when first-line treatment was either chloroquine or SP, training teachers to provide treatment (without parasitological diagnosis) was shown to be both feasible and to reduce school absenteeism and malaria deaths (Pasha *et al.* 2003; Afenyadu *et al.* 2005). However, now that WHO strongly recommends diagnosis before treatment (Test, Treat, Track), teachers need to learn how to use rapid diagnostic tests (RDTs) as well as give treatment. Encouraging experience with community volunteers and private shopkeepers suggests that this should not pose a major challenge, and an ongoing study is evaluating the impact of teacher-based diagnosis and treatment in Malawi.

### Prevention of malaria in school-age African children

A number of strategies to prevent malaria in school age children, delivered either though schools or as part of community-wide control, have been explored.

#### Insecticide-treated nets

**Fig 2:**
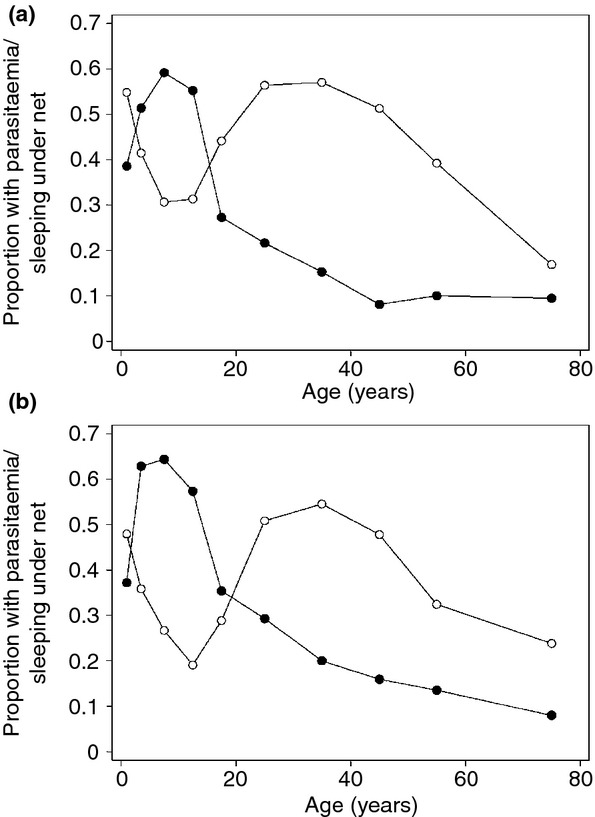
The prevalence of malaria parasitaemia by age (solid circles) and of reported use of a bednet on the previous night in Uganda. Panel (a) females, panel (b) males (Pullan *et al.* 2010, reproduced with permission).

There is strong evidence that, at the individual level, regular use of an ITN or long lasting insecticide treated net (LLIN) substantially lowers the risks of malaria (Lengeler 2004; Lim *et al.* 2011) and that an additional, indirect ‘herd’ effect is achieved when a high level of ITN coverage is obtained. Thus, most LLIN distribution programmes now aim at achieving universal coverage. As children become older and more independent, parents have less control over the time when they go to bed, where they sleep, and whether they use a net, frequently resulting in low net coverage in children in this age group. A 2009 analysis of household surveys, undertaken between 2005 and 2009 in 18 African countries, found that school-aged children were the group least likely to sleep under an ITN the previous night, with between 38% and 42% of school-aged children being unprotected (Noor *et al.* 2009). Similar low ITN usage rates have been observed among school-age children in Cameroon (Tchinda *et al.* 2012), Kenya (Atieli *et al.* 2011) and Uganda (Pullan *et al.* 2010; Nankabirwa *et al.* 2013) (Figure2). Education targeted directly at older children, for example through malaria education in schools, is likely to be the most effective way of increasing regular use of ITNs in this age group. For example, a study in Mali that followed a universal net distribution campaign found that substantially more school-age children reported using nets at the end of the malaria transmission season in schools that delivered malaria education compared to control schools (Natalie Roschnik, personal communication).

Few studies have investigated the efficacy of ITNs in school-age children specifically. An early trial in an area of low malaria transmission in central Kenya showed that, following a round of effective antimalarial treatment, sleeping under an untreated mosquito net reduced the incidence of clinical malaria, but did not reduce anaemia among children in a rural boarding school (Nevill *et al.* 1988). A reduction in the incidence of malaria was also shown in a randomised trial among 4–15 year olds on the Thai-Burmese border where malaria transmission is unstable (Luxemburger *et al.* 1994). In western Kenya, where malaria transmission is perennial and high, a community-based trial of permethrin-treated mosquito nets showed that the use of ITNs halved the prevalence of mild anaemia in adolescent schoolgirls aged 12–13 years but was less effective in preventing anaemia among schoolgirls aged 6–10 years (Leenstra *et al.* 2003). Recent cross-sectional surveys undertaken among school-age children in Somalia (Noor *et al.* 2008) and in Uganda (Pullan *et al.* 2010) suggested that net use was associated with a 71% and 43% lower risk of *P. falciparum* infection. An analysis of countrywide data from school surveys in Kenya (Gitonga *et al.* 2012) showed that ITN use was associated with a reduction in the odds of malaria infection and anaemia in coastal areas, where malaria transmission is low to moderate and among boys in western lakeshore Kenya where transmission is high.

#### Indoor residual spraying

Indoor residual spraying, the application of long acting insecticides to the walls and roofs of houses and, in some cases, public buildings and domestic animal shelters, is an effective method of malaria control. When IRS is implemented as a community-wide campaign it can achieve marked reductions in the incidence and prevalence of malaria infection in all age groups (Pluess *et al.* 2010). Repeated IRS campaigns conducted between 1955 and 1959 in the Pare Taveta region of Tanzania reduced malaria parasitaemia from 73 to 7%, and from 62 to 4% in children aged 5–9 years and 10–14 years, respectively (Draper 1960). More recently, targeted IRS conducted over 12 months in the epidemic-prone Kenyan highlands halved the monthly prevalence of asymptomatic infection in school children and reduced the incidence of clinical malaria (Zhou *et al.* 2010).

#### Reduction of breeding sites

Larval control may be effective in urban areas and in a few other epidemiological situations in Africa, such as the Kenyan highlands (Fillinger & Lindsay 2011), but it is generally not a cost effective approach to malaria control in rural areas of sub-Saharan Africa. Thus, there is likely to be little health benefit from encouraging school children to destroy potential breeding sites in school grounds, although this may help to reduce numbers of ‘nuisance’ mosquitoes.

#### Chemoprophylaxis

Chemoprophylaxis, the regular administration of anti-malarial drugs to those at risk over a sustained period of time in order to obtain persistent, protective blood levels is not generally recommended for residents of malaria endemic areas because of the threat that this will enhance drug resistance, problems with adherence and cost. However, there is compelling evidence for the benefits of chemoprophylaxis in school-age children. In an early study in Ghana, chemoprophylaxis reduced school absenteeism significantly (Colbourne 1955). In Liberia, it halved the incidence of clinical attacks in children aged 2–9 years (Björkman *et al*. 1986) and in Tanzania it had a similar impact in those 5–9 years old (Lemnge *et al.* 1997). A 2003 review of trials of malaria chemoprophylaxis in the population of malaria endemic areas reported significant health impacts in nearly all studies (Prinsens Geerligs *et al.* 2003). Most of these studies focused on young children, but in 30 of the 36 trials that examined infection rates in children over 5 years of age, reductions in malaria parasitaemia ranging from 21 to 100% were seen. A more recent review confirmed these findings (Meremikwu *et al.* 2008). At the time when malaria parasites were highly sensitive to choloquine, ‘chloroquinisation’ of schoolchildren during the peak transmission season was widely deployed in some countries in West Africa such as Senegal.

#### Intermittent preventive treatment

Intermittent preventive treatment – the periodic administration of a full therapeutic dose of an antimalarial or antimalarial combination to groups at increased risk of malaria, an approach used widely in pregnant women (IPTp) and effective in infants (IPTi) is an alternative to chemoprophylaxis. IPT is now being tested in school age children in two ways – intermittent parasite clearance in schools (IPCs) and seasonal malaria chemoprevention (SMC).

IPCs involves the administration of IPT on a periodic basis to school children to clear asymptomatic malaria infections and to aid haematological recovery during the ensuing malaria-free period. Studies which have evaluated IPCs in school-age children are summarised in Table4. The first study of IPCs (then called IPT), conducted in schools in western Kenya, showed that IPCs with SP and AQ given once a term significantly reduced malaria parasitaemia and anaemia and significantly improved sustained attention, as described above (Clarke *et al.* 2008). However, the spread of resistance to SP and AQ, and subsequent withdrawal of these drugs in many East African countries, has precluded further investigation of IPCs using these drugs in this part of Africa. Studies using alternative drugs for IPCs, including DHA-PQ, have shown a similar impact on parasitaemia and anaemia, as well as on clinical malaria (Barger *et al.* 2009; Nankabirwa *et al.* 2010; Clarke *et al.* 2013b; Nankabirwa *et al.* 2014) (Table4). If IPCs is to be deployed, the drug combination used and the timing of treatments need to be adapted to suit the local epidemiology. IPCs is likely to be most effective in settings where a high proportion of children harbour asymptomatic infections and/or where malaria is a major cause of anaemia.

**Table 4:** Summary of the results of recent trials of chemoprevention in school-age children

Study setting	Population	Type	Treatment regimen	Study drug	Protective efficacy			Source
					Clinical malaria	Malaria parasitaemia	Anaemia	
Year-round transmission								
W Kenya	6735 children aged 5–18 years; 30 schools	IPCs	Treatment once every school term (three treatments per annum)	SP + AQ	Not examined	89% (73–95%)	48% (8–71%)	Clarke *et al*. (2008)
Cote d' Ivoire	591 children aged 6–14 years; one school	IPCs	IPCs at month 0 and month 3 (two treatments per annum)	SP	Not examined	No impact	No impact	Rohner *et al*. (2010)
Uganda	780 children; three schools	IPCs	Single course of treatment PE measured after 42 days	SP	Not examined	No impact	No impact	Nankabirwa *et al*. (2010)
				SP + AQ	Not examined	48.0% (38.4–51.2%)	Mean change Hb + 0.37 (0.18–0.56)	
				DP	Not examined	86.1% (79.5–90.6%)	Mean change Hb + 0.34 (0.15–0.53)	
Uganda	740 children; one school	IPCs	Treatment once a school term (four treatments per annum)	DP	No impact	54% (47–60%)	No impact	Nankabirwa *et al*. (2014)
		IPCs	Treatment once every month (12 treatments per annum)	DP	96% (88–99%)	94% (92–98%)	40% (19–56%)	
Highly seasonal transmission								
Mali	262 children aged 5–10 years; one village	SMC	Two treatments 8 weeks apart during the malaria season: (two treatments per annum)	SP	36% (12–53%)	Not examined	Not examined	Dicko *et al*. (2002)
Mali	296 children aged 6–13 years; one village	SMC	Two treatments 8 weeks apart during the malaria season: (two treatments per annum)	SP + AS	66.6%	80.7%	59.8%	Barger *et al*. (2009)
				AQ + AS	46.5%	75.5%	54.1%	
Mali	1815 children aged 6–14 years; 38 schools	IPCs	Single treatment at end of the malaria season (one treatment per annum)	SP + AS	Not examined	99% (98%–100%)	38% (9–58%)	Clarke *et al*. (2013b)
Senegal	1000 children < 10 years old; eight villages	SMC	Two treatments given monthly towards end of malaria season: (two treatments per annum)	SP + AQ	79% (10–96%)	57% (5–81%)	41% (18–58%)	Tine *et al*. (2011)

IPCs, Intermittent parasite clearance in schools; IST, intermittent screening and treatment; SMC, seasonal malaria chemoprevention; SP, sulphadoxine/pyrimethamine; AQ, amodiaquine; AS, artesunate; DP, dihydropiperaquine.

Seasonal malaria chemoprevention involves monthly administration of IPT for up to 4 months of the year to coincide with the annual peak in malaria transmission to all subjects in the target age group without prior screening. This intervention has been highly effective in reducing the incidence of clinical malaria and anaemia in young children (Wilson 2011). In 2012, WHO recommended implementation of SMC with SP + AQ for children under 5 years of age in areas of the Sahel and sub-Sahel where transmission of malaria is highly seasonal. Although less extensively researched, and not yet recommended by WHO, there is evidence that SMC is as effective in school-age children as in the children under the age of 5 years in whom most of the initial studies were done (Tine *et al.* 2011, 2014). The greatest impact of SMC has been seen when drugs are given monthly for four consecutive months during the peak malaria transmission season, although even two annual treatments, when timed to coincide with the peak of malaria transmission, can halve the incidence of malaria in school-age children (Dicko *et al.* 2008; Barger *et al.* 2009).

#### Intermittent screening and treatment

An alternative to chemoprevention is intermittent screening and treatment (IST), an intervention in which individuals are screened periodically for malaria using a RDT and those infected (whether symptomatic or not) treated with a full course of an effective anti-malarial drug combination. The potential role of IST in malaria control has been shown by modelling (Kern *et al.* 2011) and demonstrated to be effective in the control of malaria in pregnant women (Tagbor *et al.* 2010). IST is particularly suited to areas where the use of first-line artemisinin combination therapy would be needed for preventive treatment due to resistance to other drugs or where malaria risk is low or highly focal. A recent population-based study of IST in Burkina Faso where malaria transmission is intense showed no impact on the incidence of clinical malaria in children under the age of 5 years or on malaria transmission (Tiono *et al.* 2013), and a cluster randomised trial in schools on the coast of Kenya, where transmission is low to moderate, found no impact of IST on health or cognition (Halliday *et al.* 2014). Possible reasons for the absence of an impact in these studies are the inability of most currently available RDTs to detect low-density parasitaemia and a high rate of re-infection following treatment in the areas where these studies were done. The potential for this approach to control of malaria in school-age children, especially in low or highly focal transmission areas, needs further investigation.

#### Vaccination

RTS,S/AS01, the most highly developed malaria vaccine, has shown partial protection against malaria in children vaccinated at the aged of 5–17 months or at the ages of 6, 10 and 14 weeks with protection being greater and more persistent in children in the older age group (RTS,S Clinical Trials Partnership 2011; RTS,S Clinical Trials Partnership 2012). Initial, age de-escalation studies showed that the vaccine is safe and immunogenic in school-age children (Bojang *et al.* 2005) but no large-scale trial of efficacy in school-age children has been done.

### Costs and cost-effectiveness of different malaria control strategies in children

Few data exist on the cost or cost-effectiveness of different approaches to control of malaria in school-age children, with estimates available only for IPCs and IST. The delivery of three rounds of IPCs by teachers was estimated to cost US1.88 per child per year, with drug and teacher training costs constituting the largest cost components (Temperley *et al.* 2008). A comprehensive school-based malaria prevention programme, which combined education, ITNs and IPCs cost 8.66 per year per child, with the IPCs component accounting for $2.72 per year (Natalie Roschnik, personal communication). Because of the costs of RDTs, IST is more expensive than IPCs (Drake *et al.* 2011). In terms of cost-effectiveness, the estimated cost per anaemia case averted through IPCs was estimated to be US$ 29.84 and the cost per case of malaria parasitaemia averted to be US$ 5.36 (Temperley *et al.* 2008). These estimates fall within the range of per capita costs of other malaria control strategies. Simultaneous delivery by teachers of both IPCs and deworming as part of an integrated school health package could yield economies of scope and increase cost-effectiveness.

## Conclusions

Better data are needed on the burden of malaria in school-age children to inform global policy makers and funders of the increasing importance of malaria in this age group and to ensure appropriate interactions between educational and health providers at national level. A standardised approach would improve the ability to monitor progress in this special group, and to document any changes in the risk of clinical malaria. Systems to capture episodes of clinical and fatal malaria in school-age children need not be school-based, but should summarise data for this specific risk group. The potential of serological tests to help in evaluating the burden of malaria in school-age children needs to be evaluated further. Operational research is needed to determine how best to raise awareness of the importance of malaria in school-age children and on how to improve the use of established control measures such as ITNs in this age group. Improving the malaria-relevant content of school curricula will help children to help themselves and equip them with the understanding needed to accept new approaches to control of malaria; for example, the value of blood testing for parasitological diagnosis to guide appropriate treatment.

Further clinical and modelling studies are needed to understand the potential role of drugs in preventing malaria in school-age children. Seasonal chemoprevention, IPCs and IST may all have a role to play but at present it is not clear in which settings each of these approaches might be most effective and cost effective. Mass treatment approaches are least likely to be cost effective in settings of low or highly focal transmission. However, the transmission threshold at which to introduce, or withdraw, chemoprevention will only become clear through the modelling of empirical data. The optimal characteristics of drugs for IPCs and IST are likely to include low cost, a very good safety profile, exceptional tolerability, long half life and a single dose treatment. A rigorous target product profile would help guide the development of drugs for the prevention of malaria in school-age children. If RTS,S/AS01 is licensed for use in young children, a decision that may be made in 2015/6, further studies will be needed to determine if this vaccine could be of value in the prevention of malaria in school-age children and even if this is not the case, other malaria vaccines in development may be able to do so.

On the basis of the data currently available, some recommendations can be made about the management of malaria in school-age children (Box 1) but much more needs to be learnt about how to do this more effectively (Box 2). More effective control of malaria is only one part of the drive to improve the health and education of school-age children and more work is needed on how and when to integrate malaria control strategies with other school-based programmes, for example those that deliver deworming and nutritional interventions. An increase in malaria among school-aged children can be anticipated as malaria control improves across sub-Saharan Africa. National malaria control programmes need to have in place effective strategies to deal with this new challenge and researchers need to provide the necessary evidence on which to base new control programmes.

**Box 1 Policy recommendations for the control of malaria in school-age children**

- Education about causes of malaria, its clinical features and ways of diagnosing, treating and preventing the infection should be an important part of the curriculum of all schools in areas where the school-age population is at risk of malaria infection.
- National malaria control programmes need to pay increasing attention to the problem of malaria in school-age children as the overall incidence of malaria declines and, as a consequence, the proportion of cases of malaria in older children increases.
- All school-age children resident in an area where they are at risk from malaria should sleep under an ITN.
- School-age children who develop clinical malaria must be able to recognise the nature of their illness and have easy and rapid access to reliable diagnosis and effective treatment either in their school or at a nearby health facility.

**Box 2 Six research priorities for gaining a better understanding of the challenges of malaria in school-age children**

**Epidemiology**

- Acquisition of better knowledge of the magnitude and features of malaria in school age children, especially in areas where the overall incidence of malaria is declining.

**Pathogenesis**

- Investigation of the importance of malaria as a cause of anaemia in school-age children and of how this anaemia is caused.
- Investigation of the mechanisms by which severe and uncomplicated malaria impair cognition and educational achievement.

**Treatment**

- Investigation of how effectively, and cost effectively, malaria can be diagnosed, using a rapid diagnostic test, and treated effectively by school staff in different settings.

**Prevention**

- Finding ways of improving coverage with ITNs by school-age children.
- Investigation of the comparative advantages and cost effectiveness of intermittent parasite clearance and of screen and treat programmes in the prevention of malaria in school-age children in different transmission settings and of the circumstances which favour one or other approach.
- Exploration of the potential for vaccination to prevent malaria in school-age children.
